# Prevalence rate of laboratory defined aspirin resistance in cardiovascular disease patients: A systematic review and meta-analysis

**DOI:** 10.22088/cjim.11.2.124

**Published:** 2020

**Authors:** Parvin Ebrahimi, Zeynab Farhadi, Masoud Behzadifar, Hosein Shabaninejad, Hassan Abolghasem Gorji, Masood Taheri Mirghaed, Morteza Salemi, Kamyar Amin, Roghayeh Mohammadibakhsh, Nicloa Luigi Bragazzi, Rahim Sohrabi

**Affiliations:** 1 Department of Health Services Management, School of Health Management and Information Sciences, Iran University of Medical Sciences, Tehran, Iran; 2 Health Management and Economics Research Center, Iran University of Medical Sciences, Tehran, Iran; 3 Department of Cardiology, Babol University of Medical Sciences, Babol, Iran; 4 School of Public Health, Department of Health Sciences (DISSAL), University of Genoa, Genoa, Italy

**Keywords:** Prevalence, Aspirin resistance, Cardiovascular disease, Systematic review, Meta-analysis

## Abstract

**Background:**

Cardiovascular disease (CVD) is the first cause of mortality worldwide, with all the healthcare systems facing this very challenging issue. Aspirin continues to be the major gold-standard treatment worldwide in the prevention of thrombotic disease in patients with CVD, even though not all individuals respond to antiplatelet therapy in a similar way, being resistant to aspirin. The aim of this study was to determine the prevalence of laboratory defined aspirin resistance in CVD patients worldwide.

**Methods:**

Relevant articles were identified through searching EMBASE, PubMed/ MEDLINE, ISI /Web of Science, Scopus, and the Cochrane Library, from January 2000 to February 2018. The methodological quality of the included studies was critically appraised using the Newcastle-Ottawa scale. The pooled prevalence of laboratory defined aspirin resistance was computed using the Der Simonian-Laird random-effect model.

**Results:**

We included 65 studies, with a total of 10,729 patients. The overall prevalence of laboratory defined aspirin resistance in CVD patients was 24.7% ([95%CI 21.4-28.4]. Women were found to be at increased risk of laboratory defined aspirin resistance compared to men, with an odds ratio of 1.16 [95%CI 0.87-1.54]

**Conclusion:**

Doctors and healthcare providers should pay special attention to aspirin resistance since lack of awareness could cause problems and increase mortality in these patients, if not properly treated with higher aspirin doses.

Cardiovascular disease (CVD) is the first cause of mortality worldwide, with all the healthcare systems facing this very challenging issue. The World Health Organization (WHO) estimates that 31% of the world's deaths are due to CVD, with around 17.7 million CVD-related deaths that occurred in 2015. Approximately 7.4 million of these deaths were due to heart disease and 6.7 million deaths were due to stroke (1). More than three-quarters of CVD-related deaths occur in low- and middle-income countries. The most important risk factors for heart disease and stroke are unhealthy diet, physical inactivity, tobacco and alcohol use, which lead to high blood pressure, sugar, fat, overweight and obesity (2). Platelet activation plays an important role in the development of CVD. Antiplatelet therapy prevents platelet aggregation and thrombosis, and can be used in primary and secondary prevention of CVD (3). Despite the development of next-generation drugs, aspirin continues to be the major gold-standard treatment worldwide in the prevention of thrombotic disease in patients with CVD (4). 

From a biochemical standpoint, aspirin inhibits the conversion of arachidonic acid to thromboxane A2, the main metabolite of prostaglandin synthesis, *via* cyclooxygenase (COX) (5). Even low daily aspirin doses (in the range 75-150 mg) are able to suppress biosynthesis of thromboxane, inhibiting the accumulation of platelets, and reducing the risk of CVD (6). However, aspirin does not always prevent the formation of thromboxane A2 due to failure to inhibit platelet COX (7). As such, all individuals do not respond to antiplatelet therapy in a similar way; some people suffer from thromboembolic events despite ongoing antiplatelet therapy (8, 9). The mechanism of resistance to aspirin is still unclear. Different patients may require different doses of aspirin to inhibit platelet function (10) and this calls up for a personalized treatment.

Several studies have been conducted to evaluate the rate of resistance to aspirin in CVD patients. Therefore, the aim of this study was to determine the prevalence of aspirin resistance by conducting a systematic review and meta-analysis of aspirin resistance in CVD patients worldwide. 

## Methods

The research question of the present work is the worldwide prevalence rate of laboratory defined aspirin resistance in CVD patients. This is a systematic review and meta-analysis that identified aspirin resistance studies with an assessment of its adverse effects on cardiovascular patients. There are several measurement methods to investigate platelet function test, Findings showed that the blood test is more sensitive than urine level, therefore, the present study mostly used two methods of platelet function test and verify now aspirin assay, Some studies also used platelet aggregation multiple method (11). Findings of this study were reported on the basis of the “Preferred Reporting Items for Systematic Reviews and Meta-Analyses”(PRISMA) guidelines (12). We searched different scholarly electronic databases, such as EMBASE, PUBMED/MEDLINE, ISI/Web of Science, Scopus, and the Cochrane Library, from January 2000 to February 2018. To find more potentially relevant studies, the reference list of the included studies was also hand-searched. After the search, all records were entered to the EndNote Reference Manager X8. At this point, all duplicate articles were deleted. Using the Boolean operators (AND, OR), the search strategy was performed as follows: (“platelet resistance” OR “drug resistance” OR “acetylsalicylic acid” OR aspirin OR “antiplatelet platelets” OR “aspirin resistance”) AND (“cardiovascular disease” OR “ischemic heart disease” OR **“**acute coronary syndrome**”**). A total of 2047 studies were reached from databases search, after deletion of the number 650 duplicates, 987 unrelated studies and 204 articles on the base abstract were excluded, 138 studies were included to title and abstract screening. In addition, we found 32 studies based on other sources. A total of 138 studies full texts were resumed and reviewed based on inclusion criteria. Finally, 65 studies with 10,729 participants were subjected.

Studies were included if: i) designed as cross-sectional, cohort or case-control investigations; ii) studies whose data were appropriate for the calculation of the prevalence rate; iii) patients with a proper clinically established diagnosis of CVD; and iv) peer reviewed studies published in English. Studies were excluded if: i) designed as letters to editor, editorials, commentaries, case reports or case series and reviews; ii) overlapping studies (in case of repeated/ duplicate/redundant studies, the most comprehensive ones were selected); iii) studies whose data did not allow the calculation of the prevalence rate; and iv) studies whose full-text could not be accessed

Two of the authors independently selected the studies on the basis of these criteria, and in case of disagreement, a third person was used as the referee and eventually resolved the issue through discussion.

**Data extraction: **After selecting the studies, two authors independently extracted and collected the data from the included studies: namely, the surname of the first author of the article, year of publication, country of study, number of participants in the study (based on gender, if available), type of laboratory-defined aspirin resistance,  prevalence rate, and mean age or age range of participants. Before analysis we certified the precision of the data. We revised any unequal data and adjusted accordingly.

**Assessment of methodological quality: **The methodological quality of the included studies was critically appraised using the Newcastle-Ottawa (NOS) scale (13). Three, two and five stars were assigned to the scale items based on the three domains (selection of study participants, control of confounders and outcome of interest), respectively. Based on the overall score, studies were divided into three groups: high (1-4 stars), medium (5-7 stars) and low (8-10 stars) bias.

**Statistical analysis: **The pooled prevalence of laboratory-defined aspirin resistance was computed using the Der Simonian-Laird random-effect model with its 95% confidence interval (CI) (14). To calculate the effect size (ES), the total sample size and the number of laboratory-defined aspirin resistance patients were used. The I^2^ test was used to evaluate heterogeneity between studies, which was classified as low, moderate and high (25%, 50% and 75%, respectively) (15). To assess, the role of variables such as sample size, or geographic area of studies was conducted. To ensure the stability of the results and to investigate the impact of each study on the final outcome, a sensitivity analysis was performed. To examine the effect of gender in laboratory-defined aspirin resistance, odds ratio (OR) was calculated. Also, studies were ranked based on the year of publication and cumulative meta-analysis was conducted to examine the trend of changes over time. Visual inspection of the funnel plot and Egger’s regression test were used to evaluate the publication bias (16). Figures with p<0.05 were considered statistically significant. All statistical analyses were conducted with the commercial software comprehensive meta-analysis (CMA) Version 2. 

## Results

**Figure 1:**
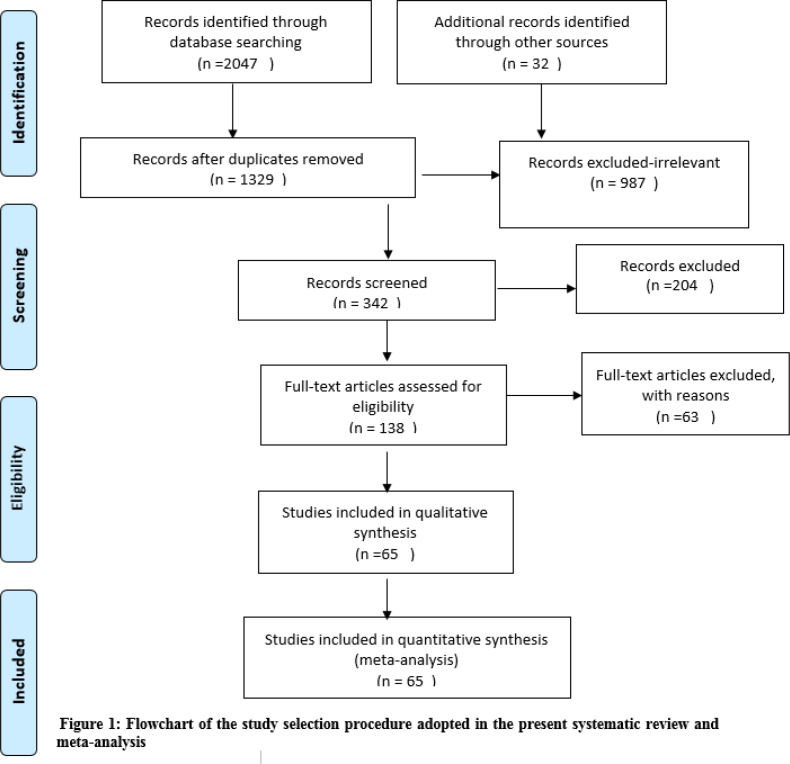
Flowchart of the study selection procedure adopted in the present systematic review and meta-analysis

After the initial search of the databases, out of a list of 2079 items, 65 studies were included and analyzed based on the above-mentioned inclusion/exclusion criteria (figure 1) (17-81). The overall number of CVD patients was 10,729. Appendix 1 shows the characteristics of the studies retained in the current systematic review and meta-analysis. 

**Appandix 1:** The characteristics of the studies

**Author**	**Year**	**Country**	**Sample size**	**Male**	**Female**	**ER**	**LL**	**UL**	**QOS**
Aksu	2014	Turkey	203	128	75	0.300	0.241	0.367	10
Akturk	2014	Turkey	134	126	19	0.164	0.111	0.237	8
Abid	2012	Tunisie	79	38	35	0.241	0.159	0.347	10
Abaci	2005	Turkey	184	96	88	0.152	0.107	0.212	10
Aksu	2009	Turkey	220	161	59	0.382	0.320	0.448	10
arslan	2015	Turkey	50	34	16	0.320	0.206	0.460	8
Aydinalp	2008	Turkey	338	168	170	0.240	0.197	0.288	8
Bach	2009	Germany	42	30	12	0.143	0.066	0.283	10
Blann	2012	UK	169	138	31	0.290	0.227	0.363	10
Çagirci	2009	Turkey	32	23	9	0.339	0.232	0.464	9
Cagirci	2010	Turkey	44	34	10	0.477	0.336	0.623	8
Acikel	2009	Turkey	97	65	32	0.299	0.216	0.397	8
Cao	2016	China	1130	872	258	0.503	0.474	0.532	10
Cao	2012	China	304	NA	NA	0.204	0.162	0.253	9
Catakoglu	2009	Turkey	100	77	23	0.140	0.085	0.223	8
Cetin	2014	Turkey	70	28	42	0.371	0.267	0.490	8
Chadha	2016	Indian	126	100	26	0.357	0.278	0.444	8
Chakroun	2007	Tunisia	191	172	19	0.157	0.112	0.216	8
Chen	2007	China	468	323	145	0.274	0.235	0.316	9
Chen	2005	China	117	88	29	0.188	0.127	0.269	10
Chen	2004	China	151	114	37	0.192	0.137	0.263	9
Cheng	2007	China	54	34	20	0.296	0.190	0.430	9
Christiaens	2002	France	50	44	6	0.200	0.111	0.333	8
Christiaens	2008	France	97	76	21	0.299	0.216	0.397	10
Chu	2010	New Zealand	314	162	152	0.477	0.336	0.623	10
Crowe	2005	Ireland	31	25	6	0.419	0.261	0.596	10
Cuisset	2009	France	136	102	34	0.014	0.091	0.209	10
Doly	2016	France	64	44	20	0.141	0.75	0.249	10
Dorsch	2007	North Carolina	94	28	66	0.298	0.214	0.398	10
Durmaz	2008	Ankara	69	54	15	0.261	o.o71	0.377	7
Floyd	2014	UK	93	32	61	0.183	0.117	0.275	10
Foussas	2009	Greece	469	344	125	0.258	0.220	0.300	10
Glauser	2009	USA	200	101	99	0.065	0.038	0.109	9
Golanski	2004	Poland.	24	24	0	0.167	0.064	0.369	8
Grove	2010	Denmark	64	49	15	0.125	0.064	0.231	9
Hiyasat	2012	Germany.	100	NA	NA	0.750	0.656	0.825	10
Hobikoglu	2005	Turkey	204	148	56	0.338	0.277	0.406	10
Hobikoglu	2005	Turkey	100	72	28	0.270	0.192	0.365	10
Ibrahim	2013	Malaysia	74	63	11	0.162	0.094	0.264	10
Kim	2011	Korea	220	162	58	o.109	0.050	0.222	10
Kim	2010	Korea	55	NA	NA	0.177	0.132	0.233	10
Kranzoeer	2006	Germany	55	NA	NA	0.455	0.329	0.586	8
Liu	2013	China	246	167	79	0.248	0.198	0.306	8
Lopez-Farre	2006	Spain	38	15	4	0.500	0.346	0.654	8
Lordkipanidze	2007	Canada	201	155	46	0.597	0.528	0.663	10
Macchi	2002	France	72	55	17	0.292	0.199	0.406	10
Manica	2012	USA	108	58	50	0.065	0.031	0.130	7
Marcucci	2006	Italy	147	116	31	0.299	0.231	0.378	10
Mirkhel	2006	USA	123	64	64	0.081	0.044	0.145	7
Narvaez	2007	Spain	268	185	83	0.164	0.124	0.213	10
Ozben	2010	Turkey	200	111	89	0.210	0.159	0.272	10
Pamukcu	2006	Turkey	234	182	52	0.190	0.126	0.277	8
Pamukcu	2007	Turkey	505	382	123	0.234	0.199	0.273	9
Poston	2005	American	225	127	98	0.298	0.242	0.361	10
Salama	2012	Egypt	50	40	10	0.220	0.126	0.355	9
Schwartz	2008	USA	184	115	69	0.038	0.018	0.078	10
Serdar	2013	Turkey	100	65	35	0.220	0.149	0.312	8
Stejskal	2006	Czech	103	66	37	0.447	0.354	0.543	10
Stolarek	2015	Poland	194	150	44	0.062	0.035	0.106	10
Tantry	2005	USA	223	131	92	0.090	0.059	0.135	10
Vivas	2011	USA	141	123	18	0.504	0.422	0.585	7
Wang	2011	UK	111	80	31	0.297	o.220	0.389	10
Ziaee	2004	IRAN	170	91	79	0.753	0.683	0.812	9
Angiolillo	2006	Italy	105	82	23	0.444	0.363	0.529	9
Pamukcu	2007	Turkey	234	182	52	0.222	0.174	0.280	9

E R =Event rate

L L=Lower limit

U L=Upper limit

Q O S=Quality of score

**Figure 2:**
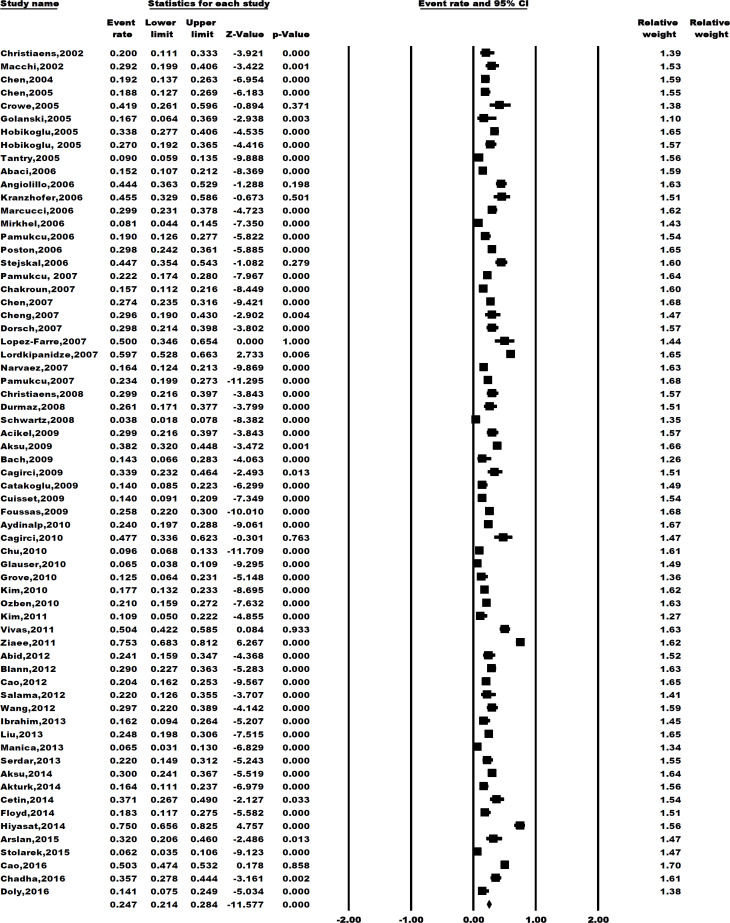
Pooled aspirin resistance prevalence rate in cardiovascular patients with its 95% confidence interval based on the Dersimonian-Laird random-effect model of the included studies in the present systematic review and meta-analysis

**Pooled prevalence of** **aspirin resistance in patients with cardiovascular disease: **The overall prevalence of aspirin resistance in CVD patients was 24.7% ([95%CI 21.4-28.4], I^2^=93.89%, p<0.001; Figure 2). 

**Results of subgroup analysis: **Based on sample size, geographic regions, year of study publication, quality of studies and gender of participants, the results of the different subgroup-analyses are shown in table 1.

**Prevalence of aspirin resistance and sample size: **Based on the sample size, the prevalence of aspirin resistance reported in 39 studies with up to 150 participants was 26.4% [95%CI 22.2-31], compared to 22.5% [95%CI 21.6-28.6], reported by 26 studies with more than 150 participants. This difference was not statistically significant (p=0.47).

**Prevalence of aspirin resistance and** **geographical background: **According to the geographic region, the prevalence in Asia was reported by 11 studies and was 27.3% [95%CI 22.4-29.4%], while the rate in Europe was available in 41 studies (25.7%, [95%CI 22.4-29.4%]). In Africa, a prevalence of 19.5% [95%CI 16.2-25.5] was found, whereas in America was of 19.1% [95%CI 10.2-32.2]. The difference in prevalence rate broken down to geographic background was statistically significant (p<0.0001).

**Prevalence of aspirin resistance and** **year of publication: **Between 2000 and 2006, 17 studies reported a prevalence rate of 25% [95%CI 19.7-31.2], whereas between 2007 and 2012, the prevalence was 24.5% [95%CI 20.2-29.2] according to 34 studies. Finally, in the years 2013-2017, the prevalence was 24.8% [95%CI 16.9-34.9]. From a statistical standpoint, the prevalence rate of aspirin resistance among CVD patients was not significant on the basis of the years of study (p=0.63).

**Prevalence of aspirin resistance and** **quality of studies: **Based on the checklist used to evaluate the quality of the studies, 5 studies with a score of 4 to 7 reported a prevalence of 42.9% [95%CI 28.9-59.1], whereas in 60 studies with a score of 8 to 10, the prevalence was 23.5% [95%CI 17.5-26.7], although this difference was not statistically significant (p=0.15).

**Prevalence of aspirin resistance and** **gender: **In 39 studies, data were suitable for calculating the prevalence of laboratory-defined aspirin resistance stratified according to gender. More in details, the prevalence in men was 23.5% [95%CI 19.5-28.0] and in women 26.9% [95%CI 22.4-31.9]. This difference was statistically significant (p<0.0001). An OR of 1.16 [95%CI 0.87-1.54] was computed (figure 3). This finding showed that women are at increased risk of laboratory-defined aspirin resistance compared to men.

**Results of cumulative meta-analysis** **for the** **prevalence of in patients with cardiovascular disease: **The studies were ranked according to the year of publication and cumulative meta-analysis was performed. The results did not change before and after this analysis, and the prevalence was 24.7% [95%CI 21.4-28.4]. Appendix 2 shows cumulative meta-analysis based on the year of publication. Studies were also ranked by sample size. The results did not change before and after the cumulative meta-analysis and the prevalence was stable. Appendix 3 shows cumulative meta-analysis based on the year of publication.

**Results of sensitivity analysis** **for the** **prevalence of aspirin resistance in patients with cardiovascular disease: **Sensitivity analysis was carried out to ensure the stability of the results of the studies. The prevalence of aspirin resistance before and after the sensitivity analysis did not change with the exclusion of each study (Appendix 4).

**Publication bias: **The Egger’s regression test results are presented in Appendix 5. Observation of the asymmetry of the funnel plot indicated that there was an evidence of publication bias (p=0.38).

**Appendix 5:**
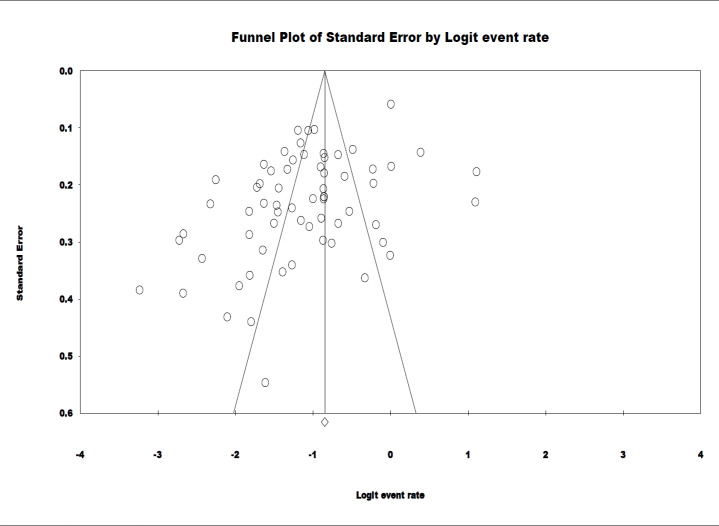
The Funnel plot of the studies included in the present systematic review and meta-analysis

## Discussion

The aim of this study was to determine the prevalence rate of laboratory defined aspirin resistance in CVD patients worldwide. The concept laboratory defined aspirin resistance has been argued since 1980s, but discussions in late literature have centralized on evidence why aspirin resistance is probably a mistake (82, 83). To the best of our knowledge, systematic search of the literature, meta-analysis and extensive statistical analyses (sub-group analysis, sensitivity analysis, cumulative meta-analysis) were the major strengths of this study. The findings showed that the prevalence of laboratory-defined aspirin resistance in CVD patients was 24.7%, with a higher rate among women. This study, pooling together different investigations reporting conflicting results, has enabled to overcome their statistical limitations and shortcomings. Some studies have, indeed, found that women have more or equal responsiveness rate to aspirin than men, being successful in controlling the COX-1 pathway, whilst other studies have shown no difference between female and male (7). According to other scholars, women would have a worse prognosis than men, whereas other studies reported that the biochemical mechanism of laboratory-defined aspirin resistance is unknown, even though female sex hormones may play an important role (84, 85). We computed an OR of 1.16 [95%CI 0.87-1.54], showing that women are at increased risk of laboratory-defined aspirin resistance compared to men.

Another important finding of the study is that, the prevalence rate is different in different regions of the world, putatively because of differences in the biological and genetic make-up of individuals. A higher prevalence was found in Asia, while the lowest rate was computed for studies carried out in America.

These findings pave the way for a personalized treatment, in that individual factors seem to affect the response to aspirin. Clinically speaking, there are some conditions known for predisposing patients to higher rate of aspirin resistance. For instance, several studies have shown that patients undergoing coronary artery bypass grafting (CABG), which results in endothelial tissue damage to the saphenous vein graft, or coronary interventions, are more likely to become resistant to aspirin, with high thrombin level and platelet activation (10, 86). This suggests that, after CABG surgery or other interventions, patients should be closely monitored and should receive plavix, alternatively, anti-thrombotic drugs. Usually, aspirin resistance after a CABG surgery persists for a short term period (22, 87). This temporal laboratory-defined aspirin resistance was in a population of patients who had withstand coronary bypass. Although no adaptation with treatment is a momentous cause of laboratory aspirin resistance, patient dependency treatment was determined in few studies (88-90). Cotter et al, have indicated no adaptation to treatment is a significant moderator of negligible consequence. It is substantial to appraise whether patients take their medicines in clinical conditions or in studies that measure the effect of prescription drugs (89, 90). According to research, another strategy to control laboratory defined aspirin resistance is the administration of vitamin D (91). Furthermore, patients not practicing enough physical activity and/or with increased blood glucose should require higher aspirin doses (92, 93).

However, despite its strengths, the present systematic review and meta-analysis suffers from some limitations, that hinder generalization of the present findings and call up for caution in interpreting results. The major drawback is given by the heterogeneity between studies and the evidence of publication bias. Another limitation is given by the methodological and quality differences among the studies. As such, further larger high-qualities studies in the field are warranted. Moreover, available study data did not allow to investigate the impact of possible risk factors associated with the prevalence of laboratory-defined aspirin resistance.

The findings of the present systematic review and meta-analysis showed that the prevalence of laboratory defined aspirin resistance in CVD patients was 24.7%. Doctors and healthcare providers should pay special attention to this, since lack of awareness could cause problems and increase mortality in these patients, if not properly treated with higher aspirin doses. It is suggested that one way to overcome the problem of laboratory defined aspirin resistance perhaps is to give the patient more medicine. However, this cannot be the result of the study, and more specific studies are required, in the way that the method of platelet related assay, the length of treatment and the amount of drug in patient have the same conditions.
